# Ultrasound-guided initial diagnosis and follow-up of pediatric idiopathic intracranial hypertension

**DOI:** 10.1007/s00247-024-05905-9

**Published:** 2024-03-20

**Authors:** Susanne Regina Kerscher, Julian Zipfel, Karin Haas-Lude, Andrea Bevot, Martin Ulrich Schuhmann

**Affiliations:** 1 Department of Diagnostic and Interventional Radiology, University Hospital of Ulm, Albert-Einstein-Allee 23, 89081 Ulm, Germany; 2 Department of Neurosurgery, Division of Pediatric Neurosurgery, University Hospital of Tuebingen, Tuebingen, Germany; 3 Department of Pediatric Neurology and Developmental Medicine, University Children’s Hospital of Tuebingen, Tuebingen, Germany

**Keywords:** Idiopathic intracranial hypertension, Non-invasive diagnosis, Optic nerve sheath diameter, Pediatric, Pseudotumor cerebri, Third ventricle diameter, Ultrasound

## Abstract

**Abstract:**

**Background:**

Idiopathic intracranial hypertension in children often presents with non-specific symptoms found in conditions such as hydrocephalus. For definite diagnosis, invasive intracranial pressure measurement is usually required. Ultrasound (US) of the optic nerve sheath diameter provides a non-invasive method to assess intracranial pressure. Transtemporal US allows imaging of the third ventricle and thus assessment for hydrocephalus.

**Objective:**

To investigate whether the combination of US optic nerve sheath and third ventricle diameter can be used as a screening tool in pediatric idiopathic intracranial hypertension to indicate elevated intracranial pressure and exclude hydrocephalus as an underlying pathology. Further, to analyze whether both parameters can be used to monitor treatment outcome.

**Materials and methods:**

We prospectively included 36 children with idiopathic intracranial hypertension and 32 controls. Using a 12-Mhz linear transducer and a 1–4-Mhz phased-array transducer, respectively, optic nerve sheath and third ventricle diameters were determined initially and during the course of treatment.

**Results:**

In patients, the mean optic nerve sheath diameter was significantly larger (6.45±0.65 mm, controls: 4.96±0.32 mm) and the mean third ventricle diameter (1.69±0.65 mm, controls: 2.99±1.31 mm) was significantly smaller compared to the control group, *P*<0.001. Optimal cut-off values were 5.55 mm for the optic nerve sheath and 1.83 mm for the third ventricle diameter.

**Conclusions:**

The combined use of US optic nerve sheath and third ventricle diameter is an ideal non-invasive screening tool in pediatric idiopathic intracranial hypertension to indicate elevated intracranial pressure while ruling out hydrocephalus. Treatment can effectively be monitored by repeated US, which also reliably indicates relapse.

## Introduction

Idiopathic intracranial hypertension is a rare disease in children with an annual incidence of 0.63–0.71 in 100,000 [1–3]. Typical symptoms include headache, vomiting, and visual disturbances. However, such symptoms are often non-specific in children [4], and may also be associated with more common conditions, such as hydrocephalus. Younger children in particular sometimes appear to have no symptoms at all [5, 6]. According to the diagnostic criteria, idiopathic intracranial hypertension is characterized by increased intracranial pressure, essentially detected by invasive lumbar puncture, with normal cerebrospinal fluid composition and magnetic resonance imaging (MRI)-based exclusion of hydrocephalus or space-occupying lesions in the absence of causes of secondary intracranial hypertension, such as venous sinus thrombosis or metabolic/immune diseases [7]. Even with the revised diagnostic criteria [8], fundoscopically diagnosed papilledema still plays a major role in establishing the diagnosis, although it has been shown that a high percentage of children with a confirmed diagnosis may not develop papilledema [9, 10]. Untreated idiopathic intracranial hypertension can lead to severe visual impairment and even blindness [11]. Therapeutic options include medical treatment, especially with acetazolamide and, in severe, therapy-refractory cases, lumbo- or ventriculo-peritoneal shunt [12, 13]. Monitoring of efficacy and duration of treatment, apart from change of symptomatology, is usually based on ophthalmologic procedures, showing, for example, a decrease in papilledema, if present [2], or, in some institutions, even repeated lumbar punctures.

Transorbital ultrasound (US) of the optic nerve sheath diameter is a highly reliable, non-invasive method for estimating intracranial pressure in children [14] and adults [15], with multiple applications [16–18]. Small pilot studies have already indicated that US optic nerve sheath diameter might be a helpful diagnostic tool to identify and follow-up children with idiopathic intracranial hypertension [19, 20]. Transtemporal US determination of the third ventricle diameter provides an estimate of the lateral ventricular width [21–25], and thus can be used to evaluate for hydrocephalus.

The aim of this study was to analyze the combined use of US optic nerve sheath and third ventricle diameters in childhood idiopathic intracranial hypertension as a first-line screening-tool to point towards a possible/likely intracranial pressure increase and exclude hydrocephalus as an underlying cause. We compared baseline optic nerve sheath and third ventricle diameter scores of patients with pediatric idiopathic intracranial hypertension to a control cohort and defined optimal cut-off values for group allocation. In addition, we serially investigated the development of optic nerve sheath and third ventricle diameters before, during, and after initial therapy, and, in the long-term, before and after relapse.

## Materials and methods

### Study design

This is a prospective observational study. Between 2018 and 2022, we enrolled pediatric patients if they underwent both a transorbital US optic nerve sheath diameter and a transtemporal US third ventricle diameter determination. Inclusion criteria for the patient (=idiopathic intracranial hypertension) group were diagnosis of idiopathic intracranial hypertension according to the revised Friedman criteria [8]: clinical symptoms of increased intracranial pressure (headache, vomiting, visual disturbance), lumbar puncture opening pressure ≥25 cmH_2_0 (≥28 cmH_2_0 if child was sedated and/or obese), without cytological cerebrospinal fluid abnormalities, and absence of hydrocephalus, space-occupying lesions or venous sinus thrombosis on MRI.

For the control group, exclusion of any disease/symptoms associated with increased intracranial pressure was mandatory: absence of clinical signs/symptoms of elevated intracranial pressure and exclusion of any disease associated with increased intracranial pressure or enlarged ventricular width.

Clinical characteristics additionally recorded included age, sex, and ophthalmoscopic findings. Patients were mixed in- and outpatients and were examined without sedation. Children >3 years of age were generally examined while awake; children <3 years of age were examined while asleep, e.g., after feeding, if necessary and possible. Prior to investigation, informed consent was obtained from parents and children old enough to understand the study. All procedures performed were in accordance with the Code of Ethics of the World Medical Association (Declaration of Helsinki). The study protocol was approved by the institutional ethics committee (180/2018BO2). The study meets the Strengthening the Reporting of Observational Studies in Epidemiology (STROBE) guidelines [26].

### Study population

Thirty-six individuals met the inclusion criteria outlined for the patient group. Thirty-two children met the control group criteria.

All individuals underwent US-based examination of the optic nerve sheath and third ventricle diameters. Individuals in the patient group were evaluated at initial presentation/diagnosis before any type of therapy was administered. The US values obtained were compared with those of the control group.

After confirmation of the diagnosis, all patients with idiopathic intracranial hypertension were assigned to therapy (medical therapy [acetazolamide, trade name: Diamox, OM Pharma Suisse SA, Villars-sur-Glâne, Switzerland, ± diuretics, trade name: Furosemid-ratiopharm, ratiopharm GmbH, Ulm, Germany] and/or repeated lumbar puncture). Twenty-seven of thirty-six patients underwent repeated US optic nerve sheath and third ventricle diameter examinations during further follow-up (over a period of 0–60 months).

### Ultrasound determination of the optic nerve sheath and third ventricle diameter

**Fig. 1:**
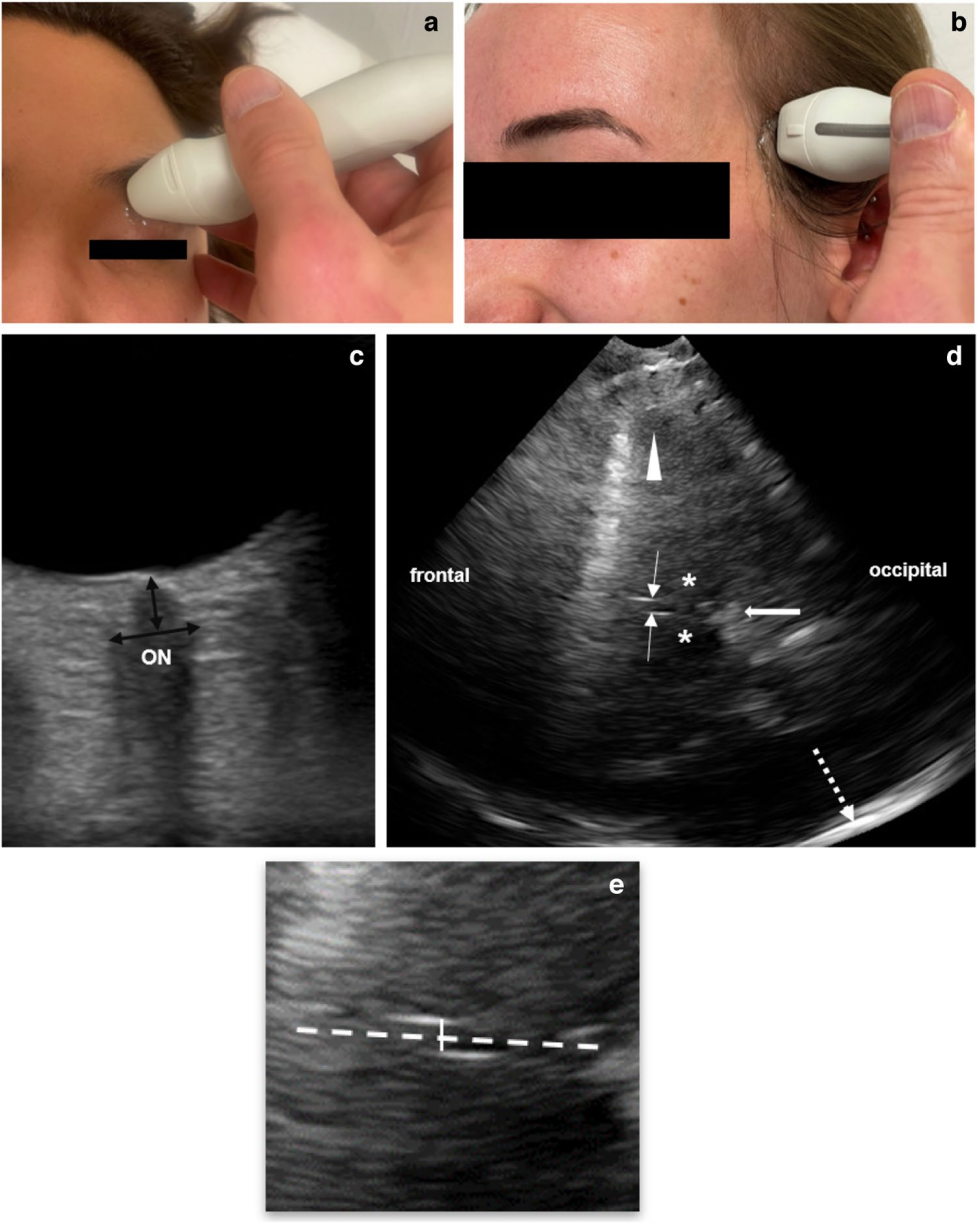
B-scan ultrasound (US) of the optic nerve sheath and third ventricle diameters in a 13-year-old girl from the control group. **a** For transorbital US examination, a 12-Mhz linear transducer is placed axially on the closed eyelid. The examiner’s hand can be placed lightly on the side of the patient’s head to improve stability. **b** For transtemporal US examination, a 1–4-MHz phased-array transducer is placed axially and pre-auricularly between the lateral canthus and the upper border of the ear. The probe orientation marker on the side of the probe should be oriented forward so that the frontal intracranial structures appear on the left side of the monitor. **c** Transorbital US of the optic nerve sheath diameter (4.71 mm) with exact region of optic nerve sheath diameter measurement (*arrows*). The optic nerve (*ON*) appears hypoechogenic. **d** Transtemporal US of the third ventricle diameter (3.1 mm), showing hyperechogenic walls of the third ventricle (*thin arrows*), bilateral, hypoechogenic-appearing thalami (*asterisks*), hyperechogenic-appearing pineal gland (*thick arrow*), ipsilateral brain parenchyma (*arrowhead*), and contralateral skull bone (*broken arrow*). **e** 1.5× magnification of a section of (**d**) showing the region of the third ventricle. Area of exact third ventricle diameter measurement (*solid line*) and the midline of the brain (*broken line*)

**Fig. 2:**
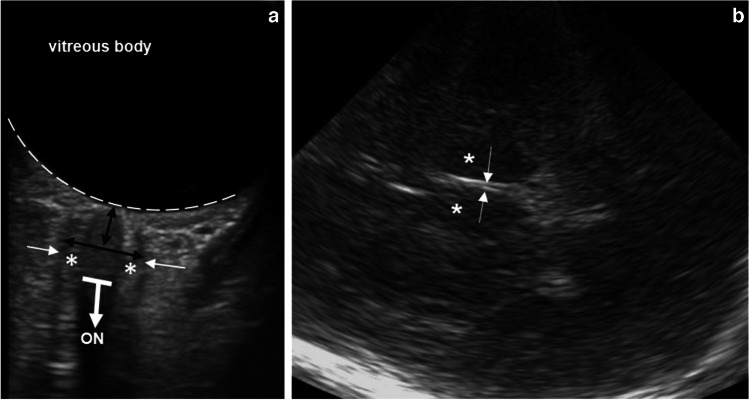
B-scan ultrasound (US) of the optic nerve sheath and third ventricle diameters in an 11-year-old girl with idiopathic intracranial hypertension. **a** Transorbital US shows an enlarged optic nerve sheath diameter (6.48 mm). The optic nerve *(ON, thick line and arrow)* appears hypoechogenic and is surrounded by the partially hyperechogenic optic nerve sheath. The wall of the optic nerve sheath appears hyperechogenic. The sheath contains the hypochogenic-appearing optic subarachnoid space, that is filled with cerebrospinal fluid and subdivided by multiple trabeculae [28], with hyperechogenic appearance (*asterisks*)*.* The outer border of the optic nerve sheath (*thin arrows*) is the exact region of optic nerve sheath diameter measurement. Area of optic nerve sheath diameter measurement, 3 mm posterior to the retina (*black arrows*), retina (*broken line*)*.* The vitreous body appears hypoechogenic. **b** Transtemporal US shows narrow third ventricle diameter (0.9 mm). Bilateral thalami (*asterisks*) and hyperechogenic walls of the third ventricle (*arrows*)

One physician (S.R.K.) with 5 years of experience in transorbital and transtemporal US performed all US investigations. The investigator was blinded to the clinical information/diagnosis. US was performed in patients in supine position, head straight, and not elevated. The optic nerve sheath diameter was examined with a 12-MHz linear transducer (Philips, Epiq 5G US system, Philips Healthcare, Best, The Netherlands) that was placed 90° to the closed eyelid in the mid-transverse plane of the globe (Fig. 1). Examination programs for transorbital B-scan US were chosen with low mechanical index values (<0.3) according to the guidelines of British Medical Ultrasound Society [27]. Machine settings, especially gain settings, were identical for all measurements. The optic nerve sheath diameter was measured 3 mm posterior and at 90° to the optic nerve. The diameter is measured by placing the marker on the outer border of each side between the hypoechogenic optic subarachnoid space and the hyperechogenic optic nerve sheath (Fig. 2). Of three measurements in axial plane per side, the mean optic nerve sheath diameter of each side and the resulting mean bi-ocular diameter were calculated.

To measure the diameter of the third ventricle, a high-resolution 1–4-MHz phased-array transducer was placed in axial plane orientation pre-auricularly on an imaginary line between the lateral canthus and the upper border of the ear over the best available bone window. A preset with the same standard setting parameters was used (penetration depths depending on age and head size between 10 cm and 16 cm, dynamic range 45–55 dB, time gain compensation, and image brightness were individually adapted). The typical, hypoechogenic midbrain formation with both cerebral peduncles was identified in every case as reference point. After tilting the probe 10–15° upwards, the third ventricle appears as a hyperechogenic double structure surrounded by the hypoechogenic bilateral thalamus (Fig. 1). The third ventricle diameter was measured three times in-between and in 90° to both hyperechogenic walls of the third ventricle and the mean diameter was calculated.

### Sample size calculation

The sample size estimate for comparison of US optic nerve sheath diameter means was calculated to be 12 patients for each group to achieve 99% power with an alpha of 0.1 (*P*<0.001). The sample size estimation for comparison of US third ventricle diameter means was calculated to be 30 patients for each group to achieve 98% power with an alpha of 1 (*P*<0.01). With an estimated loss of 10% due to exclusion criteria and data loss, the study aimed to recruit a minimum of 66 patients.

### Statistical analysis

The analyses were done using SPSS statistical software (PASW Statistics 29, IBM Corp., Armonk, NY). Data were tested for normality of distribution using Kolmogorov-Smirnov test. Parametric data were reported as means and standard deviation (SD). The independent Student’s *t*-test or Mann-Whitney *U* test was used for comparing mean values, and the dependent Student’s *t*-test or Wilcoxon sign-rank test for paired samples. Chi-quadrat test was applied for comparing nominal variables. Receiver operating characteristic (ROC) curves were created to define US optic nerve sheath and third ventricle diameter cut-off values according to Youden index calculation. Power analysis was used for sample size calculation. Statistical significance was set at *P*<0.05.

## Results

### Demographic data

A total of 68 children aged 1–7 years (mean age 9.4±4.7 years) were included. Thirty-eight (55.9%) children were male.

In the idiopathic intracranial hypertension group (*n*=36, 25 male) with a mean age of 9.7±4.4 years. Male to female ratio was 2.3:1. The control group (*n*=32, 19 males) had a mean age of 9.0±5.1 years. The difference in age and sex between the two groups was not significant (*P*>0.05).

In the idiopathic intracranial hypertension group, all patients had signs/symptoms associated with increased intracranial pressure. All individuals in the patient group underwent diagnostic lumbar puncture. Mean opening pressure was 42.1±12.2 cmH_2_0. All patients had a normal biochemical/cellular cerebrospinal fluid composition. Ophthalmologic findings at initial diagnosis were available in 34/36 patients. In 2/36, there was only oral reporting of findings, so they were excluded. 15/34 (44%) had a fundoscopic papilledema. In all patients, hydrocephalus, space-occupying lesions, and venous sinus thrombosis were excluded with MRI.

### First ultrasound-based diagnosis of idiopathic intracranial hypertension versus control cohort

US optic nerve sheath and third ventricle diameters were determined at initial presentation before any therapy. In the control group, US values were obtained after exclusion of diseases associated with increased intracranial pressure or ventricular enlargement.

**Fig. 3:**
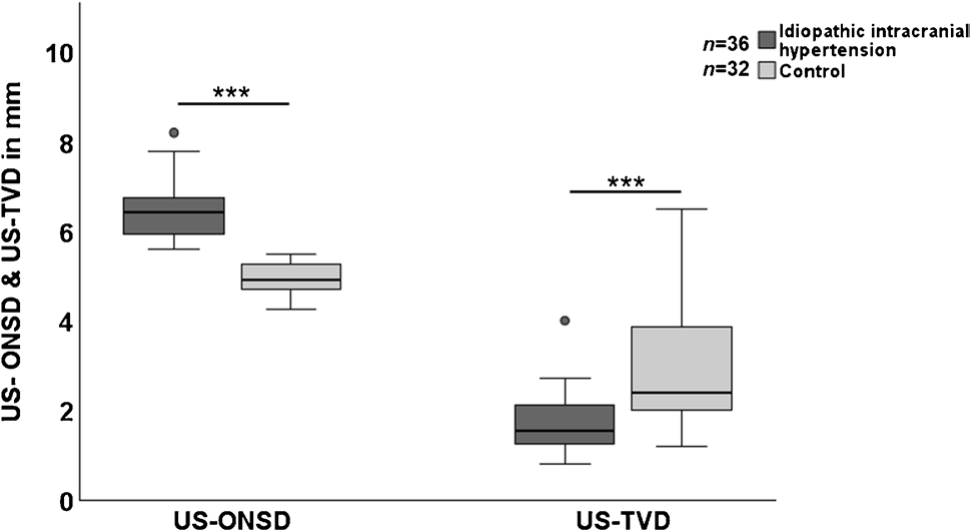
Baseline US optic nerve sheath and third ventricle diameters in idiopathic intracranial hypertension and control group. Box-plot diagram of US optic nerve sheath and third ventricle diameter in mm, ***P<0.001., *ONSD* optic nerve sheath diameter, *TVD* third ventricle diameter, *US* ultrasound

Children with idiopathic intracranial hypertension had significantly larger US optic nerve sheath diameters (mean 6.45±0.65 mm) compared to controls (mean 4.96±0.32 mm). The mean ΔUS optic nerve sheath diameter was 1.50±0.31 mm, *P*<0.001 (Fig. 3).

The mean US third ventricle diameter was significantly smaller in the idiopathic intracranial hypertension group (1.69±0.65 mm) compared with controls (2.99±1.31 mm), with a mean ΔUS third ventricle diameter of 1.30±0.25 mm, *P*<0.001.

### Optic nerve sheath and third ventricle diameter cut-off values to detect idiopathic intracranial hypertension

**Fig. 4:**
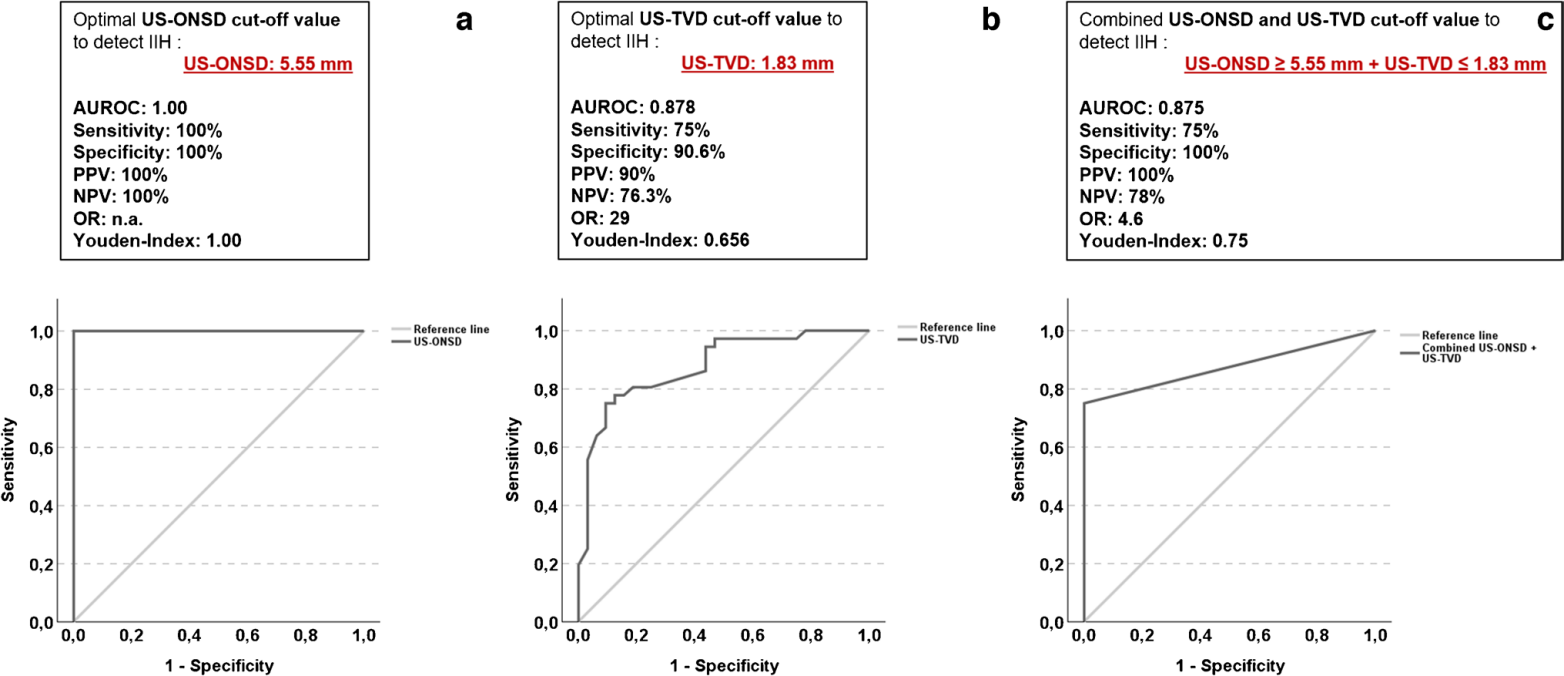
Receiver operating characteristic (ROC) analysis of ultrasound (US) optic nerve sheath diameter, US third ventricle diameter and combined use of US optic nerve sheath and third ventricle diameters to detect idiopathic intracranial hypertension in children**. a** Area under the ROC (AUROC) curve for US optic nerve sheath diameter cut-off value (≥5.55 mm). **b** AUROC curve for US third ventricle diameter cut-off value (≤1.83 mm). **c** Combined use of US optic nerve sheath and third ventricle diameter cut-off values, *n*=68. *IIH* idiopathic intracranial hypertension, *NPV* negative predictive value, *ONSD* optic nerve sheath diameter, *OR* odds ratio, *PPV* positive predictive value, *TVD* third ventricle diameter

The optimal US optic nerve sheath diameter cut-off value for predicting idiopathic intracranial hypertension in children (entire cohort, *n*=68) was 5.55 mm with an excellent diagnostic accuracy (Fig. 4). The best US third ventricle diameter cut-off value for predicting idiopathic intracranial hypertension was 1.83 mm. A combination of US optic nerve sheath diameter ≥5.55 mm and US third ventricle diameter ≤1.83 mm revealed a sensitivity of 75% and a specificity of 100% to detect or exclude idiopathic intracranial hypertension in children.

### Ultrasound-based follow-up in idiopathic intracranial hypertension

**Fig. 5:**
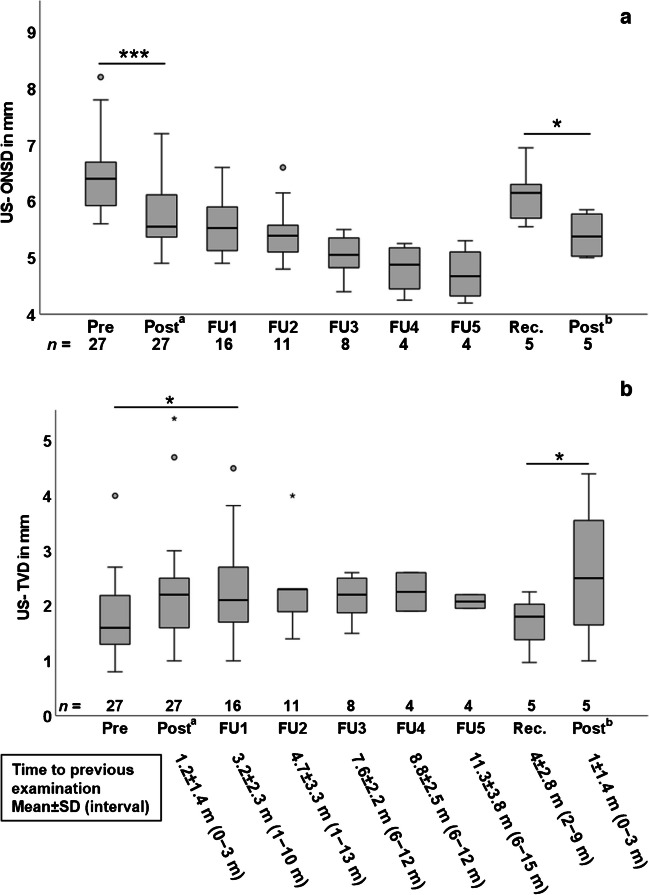
Follow-up ultrasound (US) optic nerve sheath and third ventricle diameter in children with idiopathic intracranial hypertension. Box-plot diagrams of (**a**) US optic nerve sheath diameters prior to, after treatment, and during long-term follow-up with significant decrease of optic nerve sheath diameter in 27 patients after initial treatment and in five children after relapse therapy (**b**) US third ventricle diameter prior to, after treatment, and during long-term follow-up with significant increase of third ventricle diameter in 16 patients from pre to follow-up 1 and significant increase in five children after relapse therapy. Follow-up time details are given in **b**. *P<0.05, **P<0.01, ***P<0.001. *FU* follow-up, *m* month, *ONSD* optic nerve sheath diameter, *Post*^*a*^ after initial treatment, *Post*^*b*^ after treatment for recurrence, *Pre* before initial treatment, *Rec.* recurrence, *SD* standard deviation, *TVD* third ventricle diameter

After diagnosis, therapy was initiated in all patients. Regular US optic nerve sheath and third ventricle diameter examinations were performed over a maximum follow-up period of 60 months. Follow-up examinations could not be performed in all patients for organizational reasons. Twenty-seven of thirty-six children were studied before and after therapy initiation; 16/27 received further follow-ups. In descending numbers of patients, up to five follow-up examinations were performed. Due to decreasing numbers, a statistical evaluation of the optic nerve sheath diameter was only valid in the 27 individuals before (6.44±0.66 mm) vs. 0−3 months after therapy initiation (5.78±0.58 mm), showing a significant decrease (*P*<0.001). Optic nerve sheath diameter values continued to decrease to a mean 4.7±0.49 mm during follow-up (Fig. 5 and Table 1).

**Table 1:** Ultrasound optic nerve sheath and third ventricle diameters at different study time points in children with idiopathic intracranial hypertension

US-ONSD	Mean (mm)	SD	US-TVD	Mean (mm)	SD
**Pre**	6.44	0.66	**Pre**	1.69	0.65
**Post**^a^	5.78	0.58	**Post**^**a**^	2.27	1.03
**FU1**	5.57	0.53	**FU1**	2.37	0.97
**FU2**	5.44	0.54	**FU2**	2.30	0.82
**FU3**	5.04	0.39	**FU3**	2.15	0.41
**FU4**	4.81	0.45	**FU4**	2.25	0.49
**FU5**	4.71	0.49	**FU5**	2.07	0.18
**Rec**.	6.13	0.55	**Rec.**	1.71	0.53
**Post**^b^	5.40	0.44	**Post**^**b**^	2.60	1.40

^a^After initial treatment

^b^After treatment for recurrence

*FU* follow-up, *ONSD* optic nerve sheath diameter, *Pre* before initial treatment, *Rec.* recurrence, *SD* standard deviation, *TVD* third ventricle diameter, *US* ultrasound

Clinical symptoms and papilledema (if present) regressed in 31/36 patients during initial treatment. Five of 36 children were persistently symptomatic and maintained residual papilledema; consequently, the acetazolamide dose was increased. The optic nerve sheath diameters were (non-significantly) higher in these patients than in patients without papilledema (5.90±0.37 mm vs. 5.65±0.48 mm, *P*>0.05). Two of those five patients had an initial lumbar puncture opening pressure >50 cmH_2_0 and persisting headaches under medication, so repeated lumbar punctures (with persisting intracranial pressure elevation>28 cmH_2_0) and cerebrospinal fluid drainage were performed. The optic nerve sheath diameters of these patients were 6.18 mm and 5.95 mm. During further follow-up under therapy papilledema, symptomatology, persisting intracranial pressure elevation, and the optic nerve sheath diameters (mean 4.91±0.35 mm) regressed in all five patients.

In 5/36 children, acetazolamide was gradually reduced (mean time interval after therapy initiation: 9.3±1.2 months), since papilledema (initially present in 5/5) was undetectable and children had been asymptomatic for at least 6 months. The optic nerve sheath diameter dropped from initial 6.58±0.61 to 5.8±0.38 mm, and finally to 5.1±0.25 mm at the time acetazolamide reduction was started. The mean reduction interval was 6.2±0.5 months, acetazolamide was discontinued in all patients, and the mean optic nerve sheath diameter at discontinuation had further decreased to 4.78±0.34 mm.

Another five out of 36 patients experienced a disease recurrence following reduction of medication 2−9 months after their last investigation (confirmed with lumbar puncture, mean intracranial pressure 37.3±84 cmH_2_0). In 5/5, the initial symptoms returned. Papilledema was initially present in 2/5. It disappeared under therapy and returned in both. One of five children developed new papilledema, not present at diagnosis. Therapy was intensified in all five (increase in medication), resulting in symptom relief and regression of papilledema, if present. At recurrence, these patients had a renewed increase in the optic nerve sheath diameter to 6.13±0.55 mm, which significantly decreased to 5.40±0.44 mm 0−3 months after increase in medication, *P*<0.05, (Fig. 5).

The very narrow baseline third ventricle diameter of the idiopathic intracranial hypertension cohort (*n*=27/36) had already increased by the time of the first examination (0−3 months after diagnosis and therapy initiation, see “Post^a^” in Fig. 5 and Table 1) from mean 1.69±0.65 mm to mean 2.27±1.03 mm. In 16/36, it showed a significant further increase on first follow-up (2.37±0.97 mm, *P*<0.05) compared with the baseline value for these 16 patients (1.74±0.66 mm). During all further follow-up examinations, the patients showed stable third ventricle diameter values (2.07−2.30 mm), which were overall higher than baseline of the patient group, but still below the control group (2.99±1.31 mm). The five patients who relapsed showed a considerable new decrease in the third ventricle diameter (mean 1.71±0.53 mm). After increase in medication, a renewed significant increase in the third ventricle diameter (2.60±1.40 mm) was observed (Fig. 5, Table 1).

## Discussion

In our cohort, only 44% of the children with idiopathic intracranial hypertension had initial papilledema. Although the data on papilledema in pediatric idiopathic intracranial hypertension are not uniform, our results are in agreement with some previous retrospective publications on children with this diagnosis, where only 50% [9, 29], 52% [30], or 66% [10] had initial papilledema, respectively. A further small study on secondary intracranial hypertension in children with cryopyrin-associated periodic syndrome found papilledema only in 1/6 (16.7%) patients despite intracranial pressure values between 28 cmH_2_0 and 45 cmH_2_0 [31]. Although optical coherence tomography is used more frequently to aid in the diagnosis of papilledema in children [32], it requires relatively high levels of equipment, expertise, and cooperation from the child and cannot be performed at the bedside.

The pathophysiology of idiopathic intracranial hypertension appears to be multifactorial and more complex in children compared to adults [2], where predominantly young, overweight women are affected, with a male to female ratio of 1:8.5 [33]. Our cohort had a male to female ratio of 2.3:1, similar to the results of Masri et al. with a male to female ratio of 2.1:1 [10]. Another study described different sex ratios in prepubertal (male to female ratio 8:5) vs. pubertal children (male to female ratio 5:9) [34].

Our results, together with the existing literature, suggest that in pediatric idiopathic intracranial hypertension, more males may be affected than in adults and, secondly, papilledema—as an indicator of increased intracranial pressure—seems to be present in about 50% of patients. Therefore, other more reliable non-invasive methods for diagnosis and follow-up are desirable.

Symptoms of idiopathic intracranial hypertension in children are often non-specific [4] or associated with more common conditions such as hydrocephalus. In addition, the classic diagnostic setup is based on MRI, invasive measurement of intracranial pressure [8], and fundoscopy, the latter of which has the problem of potentially low sensitivity [9, 10, 29–31]. Therefore, further diagnostic screening tools, that can quickly, reliably, and non-invasively guide suspicion towards elevated intracranial pressure and exclude hydrocephalus, are needed and useful in suspected pediatric idiopathic intracranial hypertension.

US measurement of the optic nerve sheath diameter is known to provide a non-invasive assessment of elevated intracranial pressure, as optic nerve sheath diameter correlates well with intracranial pressure values and thresholds for potentially elevated pressure are defined [15, 35, 36]. The use of US optic nerve sheath diameter in idiopathic intracranial hypertension has already been described in both adults [37] and children [19]. Its reliability in children has been demonstrated in a comparative MRI-based study [38].

Transtemporal US measurement of the third ventricle diameter is a well-established method [39], originally described in patients with neurodegenerative diseases [40]. Since the third ventricle diameter in childhood hydrocephalus reflects the lateral ventricular width (excluding rare hydrocephalus forms above the third ventricle due to foramen Monro blockage), it can be examined by proxy [21]. The reliability of this method in children has also been demonstrated in a comparative MRI-based study [23].

In our patient cohort, the mean initial optic nerve sheath diameter was 6.45±0.65 mm. This corresponds to a high probability of an intracranial pressure >27 cmH_2_O compared to a published US optic nerve sheath diameter cut-off value of 5.75 mm [35]. Due to the very narrow third ventricle diameter (1.69±0.65 mm), the working diagnosis after US screening was “significant intracranial pressure elevation without underlying hydrocephalus,” which was ultimately confirmed by subsequent diagnostics.

The herein reported US optic nerve sheath diameter values confirm findings of smaller cohort studies in children with idiopathic intracranial hypertension. One prospective study on 13 children found US optic nerve sheath diameter values between 5.3 and 8.2 mm at intracranial pressures >25/30 cmH_2_0 [19]. Aslan et al. described mean US optic nerve sheath diameter values of 6.7 mm in a prospective study on seven children [41]. Another study on eight children found initial US optic nerve sheath diameter values of 5.94±0.46 mm [20].

Irazuzta et al. described US optic nerve sheath diameter values between 3.75 and 4.9 mm in children with intracranial pressure <25 cmH_2_0 [19]; other studies published optic nerve sheath diameter cut-off values of <5.2/5.3 mm for intracranial pressure <10 mmHg [35, 42], or for a healthy control group [41], respectively. Correspondingly, our control group had a mean optic nerve sheath diameter of 4.96±0.32 mm.

Regarding the third ventricle diameter, only Sari et al. have provided data on a normal third ventricle diameter in children (maximum width of third ventricle diameter in girls 4.98±0.34 mm, 5.54±0.35 mm in boys) [43]. Our finding of a subnormal, significantly narrower third ventricle diameter in idiopathic intracranial hypertension raises the question of whether subnormal ventricle width in children is pathognomonic for the disease. Similar pediatric studies on the topic do not yet exist. Studies in adults with idiopathic intracranial hypertension have described narrow lateral ventricles in a small percentage (3.3%), but a general association between this diagnosis and very small ventricles has not been proven yet [44]. However, according to the Kelly-Monroe doctrine [45], compression of the ventricles in the context of idiopathic intracranial hypertension with increasing intracranial pressure due to venous hypertension and thus increasing periventricular cerebral blood volume is quite conceivable as an underlying mechanism, resulting in a subnormal ventricular width.

We defined cut-off values for US optic nerve sheath and third ventricle diameter to detect or exclude idiopathic intracranial hypertension in children in first-line diagnostic screening. Both optic nerve sheath and third ventricle diameter provide a high diagnostic accuracy, with elevated optic nerve sheath diameter indicating elevated intracranial pressure and subnormal third ventricle diameter excluding hydrocephalus as the underlying cause. The combined use of both parameters revealed a high diagnostic accuracy. In contrast to US optic nerve sheath diameter alone, the combination allows a clear distinction between increased intracranial pressure due to hydrocephalus (a more common cause in children) and idiopathic intracranial hypertension (a rarer cause). Several studies in the literature have established US optic nerve sheath diameter cut-off values for idiopathic intracranial hypertension, especially in adults, with excellent diagnostic accuracy comparable to our results [37, 46]. However, US third ventricle diameter cut-off values and the combination with optic nerve sheath diameters for diagnosis have not previously been published.

This is the largest study with the longest follow-up using US techniques in pediatric idiopathic intracranial hypertension. A prospective study on eight children and US optic nerve sheath diameter-based treatment monitoring was published with a maximum follow-up period of 18 months [20]. Initial optic nerve sheath diameter values were 5.94±0.46 mm and decreased, very similar to our results, to 4.59±0.12 mm. Relapse due to interruption of therapy was evident in two patients by a renewed increase in optic nerve sheath diameter.

In our patient cohort, decreasing optic nerve sheath diameter values exquisitely indicated success of therapy as evidenced by decreasing clinical symptoms, papilledema or intracranial pressure, if measured. In contrast to the rather rapid improvement of the clinical condition within weeks to a few months, the optic nerve sheath diameter levels decreased immediately, but did not normalize until follow-up 2/follow-up 3, which corresponds to a time interval of 8–38 months after the start of therapy. It is unknown if the intracranial pressure, since it was not measured in all patients, also took such a long time to normalize. Delayed regression of the elastic optic nerve sheath to normal values after long-standing extension in the sense of a hysteresis behavior is a likely explanation for the delayed normalization of the optic nerve sheath diameter [47]. A recently published retrospective study of 17 children with idiopathic intracranial hypertension confirmed the utility of US optic nerve sheath diameter as an initial diagnostic tool, but its use for follow-up was found to be limited [48]. A cystic formation of the optic nerve sheath was seen in 16/17 children which did not show a significant decrease in diameter during the course of treatment. We have occasionally made similar observations, which is reflected in the standard deviations, but the majority of our patients did not have cystic and persistently wide optic nerve sheath diameters. Such phenomena should be investigated in larger cohorts and must definitely be considered as a cause of persistent optic nerve sheath diameter enlargement. However, even in such cases, a general, albeit non-significant trend towards smaller US optic nerve sheath diameters may be indicative of treatment success.

Our study is the first to systematically investigate third ventricular width at initial presentation and during follow-up in pediatric idiopathic intracranial hypertension. Interestingly, the initially below-average third ventricle diameter behaved divergently to the decreasing optic nerve sheath diameter during follow-up since a significant increase occurred. Relapse was indicated by a renewed reduction of the third ventricle diameter with repeat increase after successful restoration of therapy. Moreover, the third ventricle diameter reached a stable level more quickly after successful treatment than the optic nerve sheath diameter (0–13 months), so that possible hysteresis effects of the third ventricle walls seem to be less pronounced.

Regarding the pathophysiology of the disease, the increased venous pressure should result in a cerebrospinal fluid reabsorption impairment via increase in cerebrospinal fluid outflow resistance [49], leading to ventricular distension. However, venous outflow impairment also results in a higher cerebral blood volume, thus higher turgor of the parenchyma, which prevents ventricular dilation. Furthermore, the increase in blood volume is compensated for by reduction of the cerebrospinal fluid volume [45], also resulting in ventricular narrowing.

One limitation of this study is, despite its prospective nature, the still small number of subjects, especially during the long-term follow-up. Larger, ideally multicenter studies might allow to derive general recommendations for diagnosis and follow-up of pediatric idiopathic intracranial hypertension with US. Another limitation is that all US investigations were performed by one (experienced) examiner. Although numerous studies have shown high repeatability and inter- and intraobserver reliability for both US optic nerve sheath [14, 50] and third ventricle diameter [23, 40], studies with multiple examiners may further support a general recommendation to use these techniques in children with idiopathic intracranial hypertension.

## Conclusions

The combined use of US-based optic nerve sheath and third ventricle diameters, taking into account the existing cut-off values, is an ideal complementary non-invasive screening tool to support the clinical suspicion of idiopathic intracranial hypertension in children by indicating increased intracranial pressure while excluding hydrocephalus. Below-average small US third ventricle diameter values may additionally strengthen the suspicion of idiopathic intracranial hypertension. By using both parameters during follow-up, treatment can be monitored easily, quickly, and reliably at the bedside.

## Data Availability

The data are not publicly available due to restrictions, e.g., their containing information that could compromise the privacy of research participants. The datasets generated during and/or analyzed during the current study are available from the corresponding author on reasonable request.
